# Assessment of Parental Awareness of Space Maintainers in Al-Madinah, Saudi Arabia

**DOI:** 10.7759/cureus.72416

**Published:** 2024-10-26

**Authors:** Reem K Naaman, Randah H Al Blowi, Maryam A Alshaikh, Abeer A Ghudaf, Ohod A Alhazmi, Abdulaziz M Alsehli, Mahmoud M Ali

**Affiliations:** 1 Preventive Dental Sciences, Taibah University, Madinah, SAU; 2 Dentistry, Taibah University, Madinah, SAU

**Keywords:** awareness, children, premature loss, primary teeth, saudi arabia, space maintainers

## Abstract

Objective:To evaluate parental awareness regarding space maintainers in Al-Madinah, Saudi Arabia, and examine variations in awareness based on parental sex and educational level.

Study design: It is a descriptive cross-sectional study.

Method: A self-administered questionnaire in Arabic was distributed to 630 parents from November 2021 to August 2022. The questionnaire assessed knowledge about the significance of primary and permanent teeth and the use of space maintainers in children.

Results:Out of the 630 distributed questionnaires,397 were completed, yielding a response rate of 63%. The study found that 242 (60.96%) of parents demonstrated a good understanding of the importance of primary teeth. However, only 78 (19.65%) respondents had satisfactory knowledge regarding the timing of primary teeth replacement. Awareness about space maintainers was notably low, with only 131 (33%) of parents having basic knowledge about them, 75 (18.89%) understanding the different types, and 101 (25.44%) and 135 (34.01%) knowing about the difficulties in consuming hard and sticky foods, respectively, while using space maintainers. Furthermore, only 69 (17.38%) of parents had personal experience with space maintainers for their children. Analysis showed that mothers were significantly more informed about oral health and space maintainers compared to fathers, but the level of education did not significantly influence awareness levels.

Conclusion: Parental knowledge and awareness about space maintainers among families in Al-Madinah are insufficient. Educational interventions may be necessary to improve understanding of space maintainers and their role in dental health for children.

## Introduction

Primary teeth play a crucial role in children’s development, aiding in speech, mastication, and guiding the eruption of permanent teeth. The exfoliation of primary teeth and the eruption of permanent teeth are normal physiological processes that occur at a specific time [1]. When primary molars are lost prematurely, it can lead to significant problems, including shifting of the surrounding teeth, space loss, and subsequent malalignment of permanent dentition [2].

Preserving primary teeth until their natural exfoliation is generally considered the best management strategy, as they serve as the most effective space maintainers in the dental arch. However, when a primary tooth cannot be saved due to progressive caries, pulp injuries, or dental trauma, premature extraction becomes necessary. The optimal approach to maintain space for the eruption of permanent teeth is the placement of a space maintainer [1].

A space maintainer can be a fixed or removable appliance designed to preserve the normal arch length after the premature loss of the primary tooth. Its primary function is to prevent complications, such as crowding, teeth impaction, crossbite formation, ectopic eruption, and discrepancies in the dental midline [3]. Although fixed space maintainers such as band-loop, crown-loop, lower lingual arch, transpalatal arch, and Nance appliances are more commonly used, various types of removable partial dentures are also utilized [4]. Pediatric dentists play a crucial role in providing dental care to young patients. They are responsible for raising parental awareness about preventive oral care and educating both pediatric patients and their parents on the importance of maintaining dental health in primary and mixed dentitions, particularly in the context of space maintainers [5].

Preventive orthodontics focuses on promoting healthy occlusion and preventing the development of malocclusion [6]. It involves taking preventative measures when the initial signs of occlusal maldevelopment are observed in primary or early mixed dentition. On the other hand, interceptive orthodontics involves interventions during the mixed dentition phase aimed at reducing the severity of malocclusion in permanent dentition [7]. Differentiating between cases that require preventive and interceptive orthodontic care is crucial for making timely referrals and minimizing the duration, complexity, and cost of treatment. Space maintenance, often achieved through the use of space maintainers, is a common preventive method to address and prevent issues associated with premature loss [5].

Parents’ knowledge and awareness of oral health significantly influence their children’s adoption of preventive oral health practices. It is anticipated that insufficient awareness among school children, parents, and primary care personnel could lead to delays in referring patients for timely interceptive interventions [5]. Therefore, it is essential to educate parents of pediatric patients about the risks of malocclusion resulting from the premature loss of primary teeth. Understanding the role of early detection and intervention can help prevent or reduce the severity of malocclusion [8].

Space maintainers are crucial for maintaining optimal tooth positioning and ensuring proper eruption of permanent teeth by creating pathways for them. In 2020, Alduraihim et al. assessed parental awareness of space maintainers in Al Kharj city, Saudi Arabia, and found that 312 (82.1%) of parents were unaware of space maintainers and had never received information about them. In contrast, only 115 respondents (30.26%) had some knowledge about space maintainers [8]. To our knowledge, similar data for Al-Madinah City, Saudi Arabia, is lacking. Therefore, the present study aims to evaluate the level of parental awareness regarding space maintainers in Al-Madinah and to explore how factors such as sex and educational level influence this awareness.

## Materials and methods

A cross-sectional descriptive observational study was conducted in Al-Madinah, Saudi Arabia, using an anonymous self-administered questionnaire. Based on a previous study and aiming for a 50% response rate (the minimum required to maximize the sample size) with a 5% margin of error and a 95% confidence interval, the target sample was calculated to be 385 subjects [8]. Parents of both genders, regardless of their education level, residing in Al-Madinah and having access to Google Forms, were invited to participate in the study.

Ethical approval for the study was granted by the Taibah University College of Dentistry Research Ethics Committee (TUCDREC), under registration number TUCDREC/19112021/UMMAli. Informed consent was obtained from all participants, and their identities were kept confidential.

The anonymous self-administered close-ended questionnaire was created using Google Forms and distributed via social media from November 2021 to August 2022. It was sent to 630 parents living in Al-Madinah. The questionnaire, adapted from a cross‐sectional study conducted in Al Kharj, was provided in Arabic and comprised 15 multiple-choice questions divided into three sections, with respondents allowed to select only one response for each question [8]. Section I includes two questions that collect demographic data from participants, including sex and education level. Section II features four questions that assess general oral health knowledge covering the importance of primary teeth, the age at which primary teeth typically shed, and the consequences of early loss of primary teeth. Section III comprises nine questions focused on parental knowledge of space maintainers, including previous experiences with their child’s use, types of space maintainers, the number of dental visits required, dietary considerations, maintenance practices, and actions to take if a space maintainer is lost or broken. The first part of the questionnaire included an introduction to the study, a statement ensuring the anonymity of respondent information, and a declaration of voluntary participation.

All analyses were conducted using the Statistical Package for Social Science (SPSS) software (version 23.0; SPSS Inc., Chicago, IL, USA). Descriptive analysis was performed to report the participant characteristics, with statistical significance set at 5% (p<0.05). Data collected through Google Forms were extracted and entered into SPSS. Frequencies and percentages of responses for each question were calculated to describe the levels of awareness. To examine the relationship between parental sex, educational level, and knowledge or awareness of space maintainers, the chi-squared test was employed.

## Results

A total of 397 parents out of 630 completed the questionnaire, yielding a response rate of 63%. Of these respondents, 241 (60.71%) were mothers and 156 (39.29%) were fathers. Regarding educational background, 267 (67.25%) of parents held a bachelor’s degree, while 130 (32.75%) had a high school degree or less (Table 1).

**Table 1:** Demographic data of the included parents

Variable		N=397 (n%)
Sex (n%)	Mother	241 (60.71 %)
	Father	156 (39.29 %)
Educational level	High school degree or less	130 (32.75%)
	Bachelor’s degree	267 (67.25 %)

Concerning parental knowledge about permanent teeth, 242 (60.96%) of parents recognized the importance of primary teeth as equal to that of permanent teeth. However, only 78 (19.65%) correctly identified that all primary teeth are replaced by age 12, reflecting a low level of knowledge. Additionally, 60 (15.11%) of parents disagreed with the statement that all permanent teeth erupt by replacing their primary predecessors. Nearly half of the participants (N=203, 51.13%) agreed that early loss of primary teeth can lead to malocclusion of permanent teeth (Table 2).

**Table 2:** Parental knowledge of permanent teeth

Section II questions		N=397 (n%)
Do you think primary teeth are as important as permanent teeth?	Yes	242 (60.96%)
	No	76 (19.14%)
	I don’t know	79 (19.90%)
By what age do you think all primary teeth will be replaced by permanent teeth?	4 years	13 (3.27%)
	6 years	231 (58.19%)
	12 years	78 (19.65%)
	18 years	5 (1.26%)
	I don’t know	70 (17.63%)
Do you think all the permanent teeth erupt by replacing their respective primary tooth?	Yes	145 (36.52%)
	No	60 (15.11%)
	I don’t know	75 (18.89%)
	Some of them	117 (29.47%)
Do you think early loss of primary teeth can lead to malocclusion of permanent teeth?	Yes	203 (51.13%)
	No	82 (20.56%)
	I don’t know	112 (28.21%)

Regarding knowledge of space maintainers, 266 (67%) of participants exhibited unsatisfactory knowledge levels as presented in (Table 3). Only 69 (17.38%) parents reported having used a space maintainer for their child following the loss of primary teeth. Knowledge about different types of space maintainers was also limited, with 256 (64.48%) of respondents having insufficient knowledge and only 75 (18.89%) providing correct answers.

**Table 3:** Parental knowledge about space maintainers

Section III questions		N=397 (n%)
Do you know what a space maintainer device is?	Yes	131 (33.0%)
	No	266 (67.0%)
Did you use a space maintainer device for your child after the loss of primary teeth?	Yes	69 (17.38%)
	No	328 (83.62%)
What are the types of space maintainer devices?	Removable	8 (2.02%)
	Fixed	58 (14.61%)
	Both	75 (18.89%)
	I don’t know	256 (64.48%)
In case your child is under treatment with a space maintainer, how often will you visit the dentist?	Every 3 months	42 (10.58%)
	Every 6 months	65 (16.37%)
	Every year	17 (4.28%)
	Once it’s broken	273 (68.77%)
Do space maintainers aid in the eruption of permanent teeth?	Yes	158 (39.80%)
	No	13 (3.27%)
	I don’t know	226 (56.93%)
Will the child be able to eat hard food, such as nuts with a space maintainer in his/her mouth?	Yes	37 (9.32%)
	No	101 (25.44%)
	I don’t know	259 (65.24%)
Will the child be able to eat sticky food, such as toffee with a space maintainer in his/her mouth?	Yes	15 (3.78%)
	No	135 (34.01%)
	I don’t know	247 (62.22%)
Do you think that space maintainers require special care with brushing?	Yes	172 (43.32%)
	No	24 (6.05%)
	I don’t know	201 (50.63%)
If the space maintainer is lost or broken, what would be the best time to visit the dentist?	Immediately	181 (45.59%)
	Anytime	26 (6.55%)
	No need to go	10 (2.52%)
	I don’t know	180 (45.34%)

When asked about dental visits, 273 (68.77%) of parents believed that a visit to the dentist was only necessary if the space maintainer was broken, while 65 (16.37%) thought visits should occur every six months. More than half of the participants (N=226, 56.93%) were unaware that space maintainers help in the eruption of permanent teeth.

A significant proportion of parents lacked awareness about the child’s ability to eat hard foods, such as nuts (N=259, 65.24%), or sticky foods (N=247, 62.22%) while using an indwelling space maintainer. Approximately 172 (43.32%) agreed that space maintainers require special brushing care, and 181 (45.59%) indicated they would visit the dentist immediately if the space maintainer was lost or broken (Table 3).

In examining the relationship between the parent’s sex and knowledge about permanent teeth, we found that mothers had significantly higher levels of knowledge across all variables compared to fathers (p<0.001) as shown in (Table 4). Similarly, mothers demonstrated significantly higher awareness regarding the use of space maintainers in all aspects except for previous experience with space maintainers used by their children (p=0.097) as detailed in (Table 5).

**Table 4:** The relationship between the parent’s sex and level of knowledge about permanent teeth ^*^p<0.05. ^**^p<0.01. ^***^p<0.001.

Section II questions		Parent sex						X^2^	P-value
		Mother		Father		Total			
		n	%	n	%	N	%		
Do you think primary teeth are as important as permanent teeth?	Yes	170	70.54	72	46.15	242	60.96	29.25***	<0.001
	No	42	17.43	34	21.79	76	19.14		
	I don’t know	29	12.03	50	32.05	79	19.90		
By what age do you think all primary teeth will be replaced by permanent teeth?	4 years	10	4.15	3	1.92	13	3. 27	26.76***	<0.001
	6 years	159	65.98	72	46.15	231	58.19		
	12 years	44	18. 26	34	21.79	78	19.65		
	18 years	3	1. 24	2	1. 28	5	1. 26		
	I don’t know	25	10.37	45	28.85	70	17.63		
Do you think all the permanent teeth erupt by replacing their respective primary tooth?	Yes	96	39.83	49	31.41	145	36.52	21.75***	<0.001
	No	42	17.43	18	11.54	60	15.11		
	I don’t know	28	11.62	47	30.13	75	18.89		
	Some of them	7	31.12	42	26.92	117	29.47		
Do you think early loss of primary teeth can lead to malocclusion of permanent teeth?	Yes	147	61.00	56	35.90	203	51.13	24.82***	<0.001
	No	43	17.84	39	25.00	82	20.65		
	I don’t know	51	21.16	61	39.10	112	28. 21		

**Table 5:** The relationship between the parent’s sex and level of knowledge about space maintainers ^*^p<0.05. ^**^p<0.01. ^***^p<0.001.

Section III questions		Parent sex						X^2^	P-value
		Mother		Father		Total			
		n	%	n	%	N	%		
Do you know what a space maintainer device is?	Yes	92	38.17	39	25.0	131	33.0	7.43**	0.006
	No	49	61.83	117	75.0	266	67.0		
Did you use a space maintainer device for your child after the loss of primary teeth?	Yes	48	19.92	21	13.46	69	17.38	2.75	0.097
	No	193	80.08	135	86.54	328	82.62		
What are the types of space maintainer devices?	Removable	6	2.49	2	1.28	8	2.02	4.99*	0.026
	Fixed	39	16.18	19	12.18	58	14.61		
	Both	52	21.58	23	14.74	75	18.89		
	I don’t know	144	59.75	112	71.79	255	64.23		
In case your child is under treatment with a space maintainer, how often will you visit the dentist?	Every 3 months	31	12.86	11	7.05	42	10.58	20.11***	<0.001
	Every 6 months	53	21.99	12	7.69	65	16.37		
	Every year	9	3.73	8	5.13	17	4.28		
	Once it’s broken	148	61.41	125	80.13	273	68.77		
Do space maintainers aid in the eruption of permanent teeth?	Yes	114	47.30	44	28.21	158	39.80	20.13***	<0.001
	No	11	4.56	2	1.28	13	3.27		
	I don’t know	116	48.13	110	70.51	226	56.93		
Will the child be able to eat hard food, such as nuts with a space maintainer in his/her mouth?	Yes	27	11.20	10	6.41	37	9.32	13.82**	0.001
	No	74	30.71	27	17.31	101	25.44		
	I don’t know	140	58.09	119	76.28	259	65.24		
Will the child be able to eat sticky food, such as toffee with a space maintainer in his/her mouth?	Yes	12	4.98	3	1.92	15	3.78	18.12***	<0.001
	No	99	41.08	36	23.08	135	34.01		
	I don’t know	130	53.94	117	75.00	247	62.22		
Do you think that space maintainers require special care with brushing?	Yes	132	54.77	40	25.64	172	43.32	38.37***	<0.001
	No	17	7.05	7	4.49	24	6.05		
	I don’t know	92	38.17	109	69.87	201	50.63		
If the space maintainer is lost or broken, what would be the best time to visit the dentist?	Immediately	121	50.21	60	38.46	181	45.59	20.27***	<0.001
	Anytime	23	9.54	3	1.92	26	6.55		
	No need to go	7	2.90	3	1.92	10	2.52		
	I don’t know	90	37.34	90	57.69	180	45.34		

Regarding the impact of parental educational level on knowledge of permanent teeth, we observed that it was not significantly related to most questions, except for one. Specifically, parents with a bachelor’s degree or higher were significantly more likely to agree that primary teeth are as important as permanent teeth compared to parents with secondary school degrees or less (p < 0.01) as presented in (Table 6).

**Table 6:** The relationship between parents’ educational level and level of knowledge about permanent teeth ^*^p<0.05. ^**^p<0.01. ^***^p<0.001.

Section II questions		Educational level						X^2^	p-value
		High school or less		Bachelor		Total			
		n	%	n	%	N	%		
Do you think primary teeth are as important as permanent teeth?	Yes	64	49.23	178	66.67	242	60.96	13.69**	0.001
	No	37	28.46	39	14.61	76	19.14		
	I don’t know	29	22.31	50	18.73	79	19.90		
By what age do you think all primary teeth will be replaced by permanent teeth?	4 years	3	2.31	10	3.75	13	3.27	8.86	0.065
	6 years	67	51.54	164	61.42	231	58.19		
	12 years	30	23.08	48	17.98	78	19.65		
	18 years	4	3.08	1	0.37	5	1.26		
	I don’t know	26	20.00	44	16.48	70	17.63		
Do you think all the permanent teeth erupt by replacing their respective primary tooth?	Yes	52	40.00	93	34.83	145	36.52	3.46	0.326
	No	19	14.62	41	15.36	60	15.11		
	I don’t know	28	21.54	47	17.60	75	18.89		
	Some of them	31	23.85	86	32.21	117	29.47		
Do you think early loss of primary teeth can lead to malocclusion of permanent teeth?	Yes	62	47.69	141	52.81	203	51.13	3.57	0.168
	No	34	26.15	48	17.98	82	20.65		
	I don’t know	34	26.15	78	29.21	112	28.21		

As shown in (Table 7), the educational level did not significantly affect awareness of space maintainers overall, with one exception. Notably, 22.31% of parents with a high school degree or less identified both removable and fixed types of space maintainers, compared to 17.23% of parents with a bachelor’s degree (p<0.05).

**Table 7:** The relationship between parents’ educational level and level of knowledge about space maintainers ^*^p<0.05. ^**^p<0.01. ^***^p<0.001

Section III questions		Education level						X^2^	P-value
		High school or less		Bachelor		Total			
		n	%	n	%	N	%		
Do you know what a space maintainer device is?	Yes	36	27.69	95	35.58	131	33.0	2.46	0.117
	No	94	72.31	172	64.42	266	67.0		
Did you use a space maintainer device for your child after the loss of primary teeth?	Yes	17	13.08	52	19.48	69	17.38	2.49	0.114
	No	113	86.92	215	80.52	328	82.62		
What are the types of space maintainer devices?	Removable	2	1.54	6	2.25	8	2.02	8.22*	0.042
	Fixed	10	7.69	48	17.98	58	14.61		
	Both	29	22.31	46	17.23	75	18.89		
	I don’t know	89	68.46	167	62.55	256	64.48		
In case your child is under treatment with a space maintainer, how often will you visit the dentist?	Every 3 months	15	11.54	27	10.11	42	10.58	2.56	0.465
	Every 6 months	16	12.31	49	18.35	65	16.37		
	Every year	5	3.85	12	4.49	17	4.28		
	Once it’s broken	94	72.31	179	67.04	273	68.77		
Do space maintainers aid in the eruption of permanent teeth?	Yes	43	33.08	115	43.07	158	39.80	5.55	0.063
	No	7	5.38	6	2.25	13	3.27		
	I don’t know	80	61.54	146	54.68	226	56.93		
Will the child be able to eat hard food, such as nuts with a space maintainer in his/her mouth?	Yes	14	10.77	23	8.61	37	9.32	0.63	0.730
	No	31	23.85	70	26.22	101	25.44		
	I don’t know	85	65.38	174	65.17	259	65.24		
Will the child be able to eat sticky food, such as toffee with a space maintainer in his/her mouth?	Yes	6	4.62	9	3.37	15	3.78	0.55	0.761
	No	42	32.31	93	34.83	135	34.01		
	I don’t know	82	63.08	165	61.80	247	62.22		
Do you think that space maintainers require special care with brushing?	Yes	53	40.77	119	44.57	172	43.32	1.19	0.551
	No	10	7.69	14	5.24	24	6.05		
	I don’t know	67	51.54	134	50.19	201	50.63		
If the space maintainer is lost or broken, what would be the best time to visit the dentist?	Immediately	56	43.08	125	46.82	181	45.59	0.79	0.852
	Anytime	8	6.15	18	6.74	26	6.55		
	No need to go	4	3.08	6	2.25	10	2.52		
	I don’t know	62	47.69	118	44.19	180	45.34		

## Discussion

Dental caries is highly prevalent among the Saudi population and is considered a significant health problem in Saudi Arabia [9,10]. Early studies from 1998 indicated that 87% (N=240) of six-year-old children in Al-Madinah had caries, with a mean decayed, missing, and filled teeth (DMFT) score of 6.4 in primary dentition [11]. A more recent study in 2016 found a similar prevalence rate of 86% (N=147) in the same age group in Al-Madinah, with a reduced DMFT score of 4.9 in primary dentition [12]. The high incidence of dental caries in this region frequently leads to the premature loss of primary molars. A study conducted in the Eastern Province of Saudi Arabia revealed that 51% (N=156) of the children experienced premature loss of primary teeth due to caries [13]. Common complications arising from caries include extensive carious cavities and pulp inflammation, which account for 92.6% (N=211) of premature loss of primary teeth in children older than six years [14].

Early loss of primary molars can lead to various complications, including arch length discrepancies, increased crowding severity, midline discrepancies, and buccal segment shifts [15]. The findings of this study reveal a relatively good level of awareness among parents about the importance of primary teeth, with 60.96% (N=242) recognizing their value as equal to that of permanent teeth. However, nearly half of the parents (N=194, 48.87%) were unaware that early loss of primary teeth can lead to malocclusion in permanent teeth. This is consistent with another study, where 42.9% of the parents (N=222) believed that early loss of primary teeth would cause no harm [16].

Only a few studies have reported on the level of knowledge regarding the use of space maintainers among Saudi parents [5,8,16,17]. In our study, only 69 parents (17.38%) had experience with space maintainers, and there was minimal knowledge about their use, types, and dietary restrictions associated with them. Similar findings were reported in studies by Linjawi et al., Alduraihim et al., and Alshammari et al. in other regions of Saudi Arabia [5,8,16]. The low level of knowledge about space maintainers may be linked to their insufficient use by dental practitioners in Saudi Arabia. For instance, a study conducted in Dammam reported that despite a high prevalence of premature loss of primary teeth (N=156, 51%), only five children (1.6%) were wearing space maintainers upon examination [13]. Additionally, another study found that 85 practitioners (35.5%) in Riyadh rarely or never used space maintainers in their clinics [18]. Notably, one significant reason for the underuse of space maintainers was parental refusal, which underscores how inadequate knowledge contributes to their limited application in clinical practice [18].

Space maintainers require special care, close follow-up, and regular maintenance due to potential issues such as immersion in soft tissues, interference with the eruption of permanent teeth, susceptibility to fracture, and plaque accumulation, which can lead to caries and gingival inflammation [15,19]. More than half of our respondents were unaware of the necessary actions if space maintainers were broken, and the need for special brushing care. This contrasts with the findings of Linjawi et al. who reported a better level of awareness regarding these aspects [5]. Additionally, our study revealed very low levels of knowledge among parents about the importance of regular dental visits every six months for children with space maintainers, compared to other studies that reported satisfactory levels of knowledge [17].

Interestingly, the level of knowledge about permanent teeth and space maintainers was more strongly related to the parent’s sex than to the educational level. Generally, mothers demonstrated better knowledge compared to fathers, which aligns with findings by Alshammari et al., who also observed higher awareness among female parents regarding space maintainers [16].

Given these insights, it is crucial to develop public awareness programs in Al-Madinah that target all societal segments irrespective of education level and sex. For example, community workshops can educate parents and dental professionals on the importance of space maintainers, while school-based initiatives can inform children and families about dental health. Public awareness campaigns using social media and print materials will highlight their benefits. Lastly, incentive programs encouraging families to seek dental consultations can further raise awareness and improve children’s dental health outcomes.

The findings can directly inform dental practitioners about the gaps in parents' knowledge of space maintainers, allowing them to focus their educational efforts during consultations. However, this study has some limitations; it utilized a convenience sample, and the reliance on an online survey may restrict the generalizability of the results.

## Conclusions

Parents of young children living in Al-Madinah, Saudi Arabia, exhibit very low levels of knowledge and awareness about space maintainers. While female parents demonstrated higher awareness compared to male parents, parental education levels did not correlate significantly with knowledge about space maintainers. These findings underscore the urgent need for targeted educational programs aimed at both parents and children to improve understanding and utilization of space maintainers.
